# Integrative Transcriptomic Network Analysis of Butyrate Treated Colorectal Cancer Cells

**DOI:** 10.3390/cancers13040636

**Published:** 2021-02-05

**Authors:** Saira R. Ali, Ayla Orang, Shashikanth Marri, Ross A. McKinnon, Robyn Meech, Michael Z. Michael

**Affiliations:** 1 Flinders Health and Medical Research Institute–Cancer Program, Flinders University, Bedford Park, SA 5042, Australia; saira.ali@unisa.edu.au (S.R.A.); ayla.orang@unisa.edu.au (A.O.); shashikanth.marri@flinders.edu.au (S.M.); ross.mckinnon@flinders.edu.au (R.A.M.); robyn.meech@flinders.edu.au (R.M.); 2 Flinders Centre for Innovation in Cancer, Department of Gastroenterology and Hepatology, Flinders Medical Centre, Bedford Park, SA 5042, Australia

**Keywords:** microRNAs, colorectal cancer, butyrate, histone acetylation, systems biology

## Abstract

**Simple Summary:**

High-fiber diets are known to protect against colorectal cancer (CRC), largely through the influence of butyrate, which is generated by the colonic microbiota. To better understand how dietary butyrate prevents colorectal cancer, a systems biology approach was used to define the transcriptomic responses of butyrate-treated CRC cells. Butyrate altered the expression and/or splicing of thousands of genes. Through the integration of microRNA and mRNA datasets, molecular interaction networks were generated that identified key components of the butyrate response and facilitated bioinformatic predictions of butyrate-induced changes in cellular activity. Moreover, two butyrate-regulated microRNAs identified through this analysis were shown to enhance the effect of butyrate on cellular proliferation and apoptosis. These results help create a framework for identifying novel drug targets that may act in concert with histone deacetylase inhibitors, such as butyrate, to prevent or treat cancers.

**Abstract:**

Diet-derived histone deacetylase inhibitor (HDACi), butyrate, alters global acetylation and consequently global gene expression in colorectal cancer (CRC) cells to exert its anticancer effects. Aberrant microRNA (miRNA) expression contributes to CRC development and progression. Butyrate-mediated modulation of microRNA (miRNA) expression remains under-investigated. This study employed a systems biology approach to gain a comprehensive understanding of the complex miRNA-mRNA interactions contributing to the butyrate response in CRC cells. Next-generation sequencing, gene ontology (GO) and pathway enrichment analyses were utilized to reveal the extent of butyrate-mediated gene regulation in CRC cells. Changes in cell proliferation, apoptosis, the cell cycle and gene expression induced by miRNAs and target gene knockdown in CRC cells were assessed. Butyrate induced differential expression of 113 miRNAs and 2447 protein-coding genes in HCT116 cells. Butyrate also altered transcript splicing of 1589 protein-coding genes. GO, and pathway enrichment analyses revealed the cell cycle to be a central target of the butyrate response. Two butyrate-induced miRNAs, miR-139 and miR-542, acted cooperatively with butyrate to induce apoptosis and reduce CRC cell proliferation by regulating target genes, including cell cycle-related *EIF4G2* and *BIRC5*. *EIF4G2* RNA interference mimicked the miR-139-mediated reduction in cell proliferation. The cell cycle is a critical pathway involved in the butyrate response of CRC cells. These findings reveal novel roles for miRNAs in the cell cycle-related, anticancer effects of butyrate in CRC cells.

## 1. Introduction

Colorectal cancer (CRC) is a common cause of cancer-related deaths worldwide [1]. Although a small proportion of CRC cases develop from genetic factors, most cases develop sporadically and are linked to environmental and lifestyle factors, such as diet [2,3]. The development and progression of CRC are associated with epigenetic alterations such as altered histone modification patterns, DNA methylation and dysregulated expression of non-coding RNAs, including microRNAs (miRNAs) [4,5,6,7,8].

One proposed mechanism by which diet can influence CRC risk is the production of a short-chain fatty acid, butyrate, by fermentation of dietary fiber in the gut. High levels of luminal butyrate are known to protect against CRC. Consistent with this protective effect, butyrate treatment of CRC cells regulates the cell cycle to reduce proliferation and induce apoptosis [9,10]. Butyrate functions as a histone deacetylase inhibitor (HDACi) and may exert its anticancer properties by altering the expression of both protein-coding and non-coding genes [11,12]. Interestingly, butyrate has been shown to alter gene expression in CRC cells (and colon stem cells) in a manner that suppresses growth, yet normal colonocytes appear resistant to this response [13,14]. This effect, termed the butyrate paradox, is proposed to involve differences between normal and cancerous colon cells in differentiation status, the presence/absence of oncogenic driver mutations, and metabolic parameters, including the ability of normal colonocytes to metabolize butyrate [15,16,17].

In CRC cells, there is increasing interest in the ability of butyrate to regulate miRNAs. miRNAs are small non-coding RNAs that post-transcriptionally regulate the expression of mRNAs by reducing stability and/or suppressing translation. The overlap of cell growth and survival pathways that are regulated by miRNAs and by butyrate in CRC cells suggests that butyrate may exert some of its anticancer effects through, or in cooperation with, miRNAs [12].

Microarray profiling in CRC cells has shown that butyrate can regulate several hundred miRNAs, including those with known functions, but also many that remain uncharacterized [12]. Moreover, butyrate may either increase or decrease the levels of specific miRNAs. As an example, oncogenic cluster members miR-17, miR-20a, miR-20b, miR-93, miR-106a and miR-106b (from clusters miR-17~92a, miR-106a~363, and miR-106b~25), which are normally upregulated in CRC, were decreased by exposure to 1 mM butyrate in a CRC cell line [12]. The butyrate-mediated reduction of miR-17~92 cluster members resulted in increased expression of their tumor suppressor target genes, including PTEN, BCL2L11 and CDKN1A, which are involved in inhibiting cell growth pathways and promoting apoptosis and cell cycle arrest [18]. There is also evidence for functional cooperation between miRNA activity and butyrate responses in CRC: in particular, the manipulation of the tumor suppressor miRNA, miR-18a (miR-17–92 cluster) in combination with butyrate treatment was shown to enhance the anticancer properties of 2.5 mM butyrate in CRC cells [19].

These studies suggest important roles for miRNAs in the butyrate response; however, an understanding of how butyrate alters the global CRC transcriptome and how post-transcriptional gene regulation controls cell behavior is still lacking. This study employed a systems biology approach to gain a comprehensive understanding of the complex miRNA: mRNA interactions involved in the butyrate response in CRC cells. Whole transcriptome analysis, including small RNA-sequencing, was used to identify the effects of butyrate on gene expression in CRC cells. Subsequently, integrative network and pathway analyses were used to identify key miRNA-target interactions within signaling pathways that may contribute to the anticancer effects of butyrate. Two miRNA: mRNA pairs identified by the analysis were shown to control CRC growth and survival alone and cooperatively with butyrate. These data show the utility of a systems approach in uncovering novel mediators of the butyrate effect in CRC cells.

## 2. Results

### 2.1. Identification of Butyrate-Regulated Protein-Coding Genes and miRNAs

**Figure 1:**
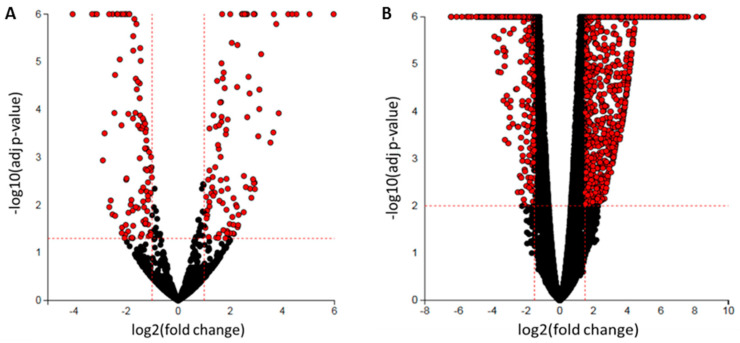
Volcano plots representing the differential expression of butyrate responsive miRNAs and protein-coding genes in HCT116 colorectal cancer (CRC) cells. The *x*-axis represents the differential expression (log2-fold change (FC)), and the *y*-axis represents the significance (–log10 (*p*-value)): (**A**) small RNA sequencing was performed to determine differential miRNA expression. Selection criteria: log2FC ≤ −1 or log2FC ≥ 1 and adj *p*-value < 0.05, (**B**) Total RNA sequencing was performed to determine differential protein-coding gene expression. Selection criteria: log2FC ≤ −1.5 or log2FC ≥ 1.5 and adj *p*-value < 0.01. Statistically significant differentially expressed miRNAs or protein-coding genes are colored in red, while black dots show those with no significant change. The plot was generated using the Advaita iPathway Guide tool [20].

To investigate differentially expressed protein-coding genes and miRNAs regulated by butyrate in CRC cells, next-generation sequencing was performed. Small RNA-seq analyses revealed 113 butyrate-regulated miRNAs (50 downregulated and 63 upregulated) (Figure 1A). Total RNA-seq analyses revealed 2447 butyrate-regulated protein-coding genes (1110 downregulated and 1337 upregulated) (Figure 1B). QIAseq Targeted RNA Panel analysis was employed as a complementary method to Illumina RNA-seq. Correlation analysis indicated good reproducibility between Illumina RNA-seq and QIAseq panel analyses with a Pearson’s correlation coefficient *r =* 0.9396 (Figure S1). Differentially expressed miRNAs and mRNAs are shown in Tables S1 and S2, respectively. The differentially expressed genes were investigated for their potential roles in the butyrate response of CRC cells through network and pathway analyses.

### 2.2. Butyrate Alters RNA Processing and Transcription Factor Activity

#### 2.2.1. Butyrate Influences Transcript Splicing

**Figure 2:**
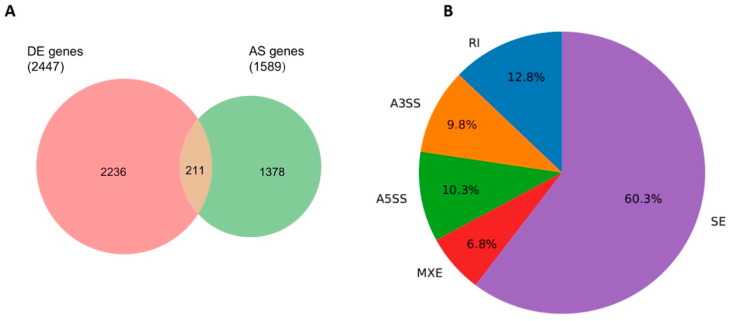
Replicate multivariate analysis of transcript splicing (rMATS). (**A**) Venn diagram comparing differentially expressed (DE) genes (padj < 0.05) and alternatively spliced (AS) genes (FDR < 0.05 or PSI > 0.1). (**B**) Breakdown of average percentages of rMATS splicing changes (events) detected between butyrate dosage of 0 mM and 2.5 mM by type of event (SE = skipped exon, RI = retained intron, MXE = mutually exclusive exon, A5SS = Alt 5 splice site, A3SS = Alt 3 splice site).

To identify changes in alternative transcript splicing induced by butyrate, replicate multivariate analysis of transcript splicing (rMATS) was performed using RNA-seq data, with a focus on protein-coding genes. Altered splicing was not reliant on differential expression, nor did most differentially expressed genes show altered splicing. Regardless, the comparison between differentially expressed genes and alternatively spliced genes revealed that 211 genes were affected by both processes after 2.5 mM butyrate treatment in HCT116 cells (Figure 2A). rMATS splicing changes indicated that exon skipping was the predominant splicing event (60.3%; FDR < 0.05) occurring in these CRC cells (Figure 2B).

#### 2.2.2. Butyrate Alters Transcription Factor Activity

To seek correlations between transcription factor (TF) activity and altered expression of protein-coding genes, ChEA3 was used to identify TFs that were differentially expressed in the RNA-seq dataset. TF data are compiled in Table S3. 1632 TFs were identified, of which 189 were differentially expressed. Seven of the top ten ChEA3-ranked TFs in this list were repressed by butyrate: FOXM1, CENPA, ZNF367, E2F7, E2F1, HMGA2, and MYBL2. The genes regulated by these TFs were also mostly downregulated, indicating that altered TF activity affects the expression of downstream genes. TransmiR was used to identify miRNAs that are predicted to be regulated by the differentially expressed TFs. Examining the same seven TFs, TransmiR analysis identified downregulated miRNA target genes for E2F7 (3/3 miRNAs), a largely downregulated miRNA gene list for FOXM1 (7/10 miRNAs), and equally up and downregulated miRNAs downstream of E2F1 and MYBL2 (Table S3).

### 2.3. Butyrate-Regulated Protein–Protein Interaction Network Analysis

**Figure 3:**
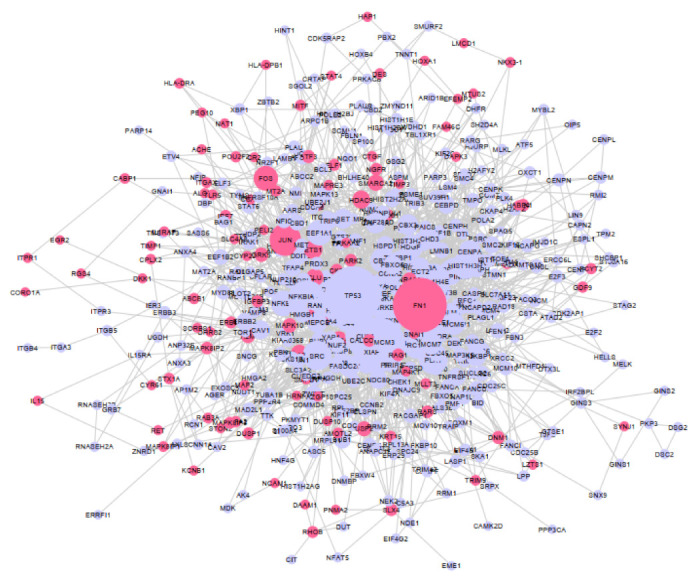
Functional enrichment analysis and network construction. Protein–protein interaction (PPI) network analyzed using NetworkAnalyst and constructed using Cytoscape software. Pink nodes represent the upregulated protein-coding genes, and purple nodes represent the downregulated protein-coding genes. Solid gray lines are edges and represent direct protein–protein interactions between two nodes. The size of the nodes is proportional to the number of interactions with other nodes, i.e., degree value.

To identify genes that may play key roles in the butyrate response, a protein–protein interaction (PPI) network was generated from the list of butyrate-regulated protein-coding genes and the major hubs identified. The differentially expressed gene (DEG) list was filtered to remove mRNAs with low expression in both untreated and butyrate-treated samples, leaving 1623 genes (1026 downregulated and 597 upregulated by butyrate). The PPI network was created using NetworkAnalyst and Cytoscape. In total, 507 DEGs whose protein product had degree ≥ 1 were identified for network visualization and further investigation (Figure 3 and Table S4). The degree value for a protein indicates its number of connections to other proteins, and nodes with the greatest degree value (most connections) are considered central hubs within the network. The PPI network analysis identified several hub nodes (greatest degree value) regulated by butyrate, with the top 3 hubs including p53 with degree 80 and KIAA0101 and FN1 with degree 75 (Table S4).

### 2.4. Functional Gene Ontology (GO) and KEGG Pathway Enrichment Analysis of Butyrate Regulated Genes

**Figure 4:**
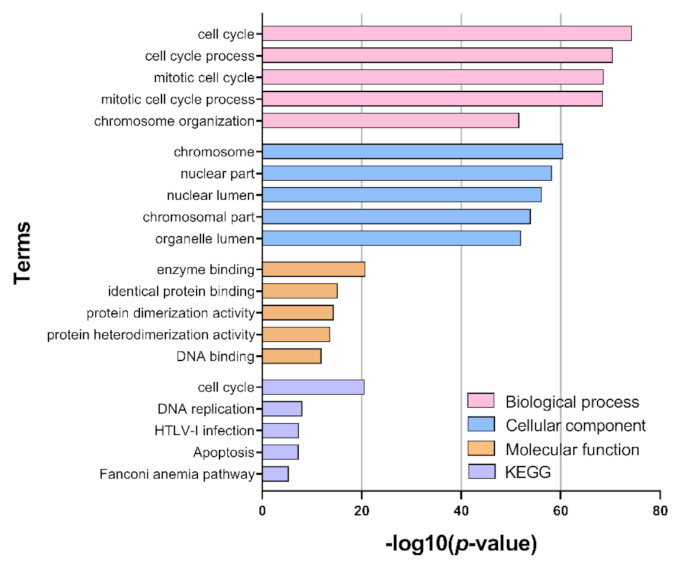
Functional enrichment analysis. Bar plot depicts the top 5 enriched gene ontology (GO) terms within categories: biological process, cellular component, molecular function and KEGG, as identified after performing ClueGO enrichment analysis in Cytoscape with butyrate responsive genes. *Y*-axis represents the GO term, and the *X*-axis represents the enrichment significance (−log10 (*p*-value)), respectively.

Functional GO and pathway enrichment analysis of differentially expressed genes was used to further characterize the butyrate response in CRC cells (Figure 4). ClueGO was used to identify the enriched GO terms for each of the following classifications: biological processes, cellular compartments, molecular functions. The most enriched terms within the biological processes classification were related to cell cycle and division. Similarly, enriched terms within the cellular compartments classification were associated with chromosomal and nuclear compartments, and enriched terms within the Molecular Functions category were associated with DNA and protein activity. KEGG pathway analysis revealed enrichment in the cell cycle and DNA replication pathways for butyrate-regulated genes.

### 2.5. Integrative Network Construction Using miRNA Target Prediction

**Figure 5:**
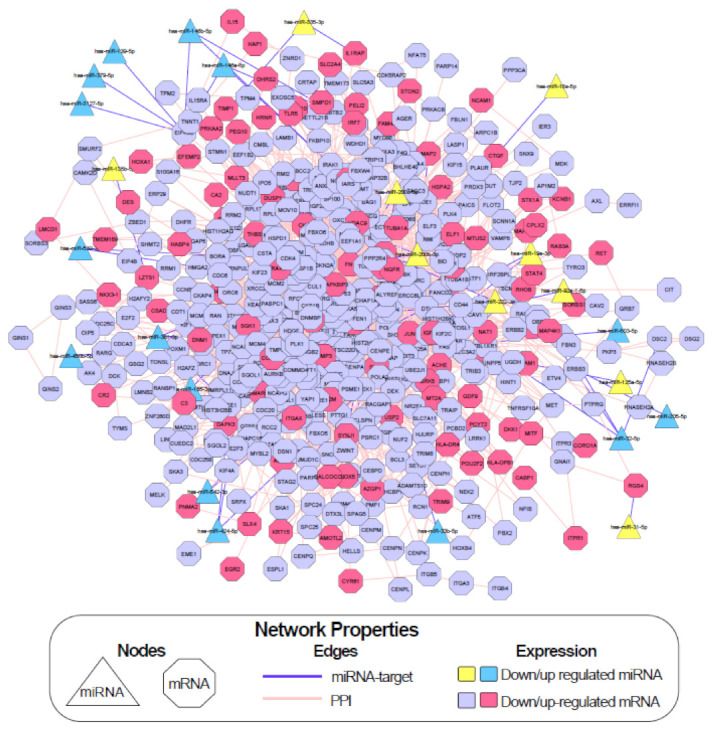
Butyrate-regulated integrative miRNA-mRNA network constructed using Cytoscape based on interactions between miRNA and target protein-coding genes and PPI. Refer to the key for node information and expression profiles. The color of the node represents the expression changes due to 2.5 mM butyrate treatment, and the shape represents the type of molecule for each node. Solid lines are edges and represent direct interactions between two nodes.

In order to identify key miRNA and mRNA gene interactions regulated by butyrate, a miRNA-mRNA network analysis incorporating PPI was performed using Cytoscape (Figure 5). The differentially expressed miRNA gene lists (Section 2.1) were filtered to remove genes with low expression in both untreated and butyrate-treated samples, resulting in 77 butyrate-regulated miRNAs (38 downregulated and 39 upregulated). Predicted and experimentally validated mRNA targets of these miRNAs were identified using a combination of online target prediction programs and validated target databases, including TargetScan Human Release 7.0 [21], miRDB [22], DIANA Tools microT-CDS [23], miRTarBase [24] and miRecords [25]. A total of 52 miRNA-mRNA target pairs were revealed in the miRNA-mRNA integrative network incorporating PPI; of these interactions, 16 involved experimentally validated targets, while 36 were predicted targets (Table S5). The network had a total of 532 nodes, including miRNAs, mRNA targets of these miRNAs, and also mRNAs that were not miRNA targets but were differentially expressed (Figure 5).

### 2.6. Investigation of Key miRNA-mRNA Interactions Involved in Cell Growth and Death Pathways in the Butyrate Response

The pathway and GO term enrichment results were used to filter the list of predicted miRNA-mRNA interactions for further analysis. The “cell cycle” pathway was highly enriched in KEGG pathway analysis, and butyrate has previously been reported to regulate cell growth and death pathways, including the cell cycle and apoptosis. (Figure 4). Thus, differentially expressed protein-coding genes that mapped to the “cell cycle” in the pathway analyses (or to cell cycle-related terms in the GO analysis) were collated for network analysis. This produced a list of 215 cell cycle-related protein-coding DEGs. To elucidate the role of miRNA-mRNA interactions in the butyrate response, predicted and validated miRNA-mRNA interactions were identified for these differentially expressed genes. We considered only interactions where the butyrate-induced expression change for the miRNA and its target mRNA were negatively correlated. The list was further refined by identifying miRNAs previously shown to function as tumor suppressors with potential oncogenic target genes involved in CRC and/or other cancer types. From this list (Table 1), two anti-correlating miRNA: mRNA pairs were selected for further investigation: miR-139: *EIF4G2* and miR-542: *BIRC5*. *EIF4G2* was prominent in the integrated network (Figure 5), while miR-542 strongly inhibits cell proliferation and *BIRC5* (survivin) is an important anticancer drug target [26].

### 2.7. Validation of the Butyrate Effect on miRNA and mRNA Target Gene Expression

**Figure 6:**
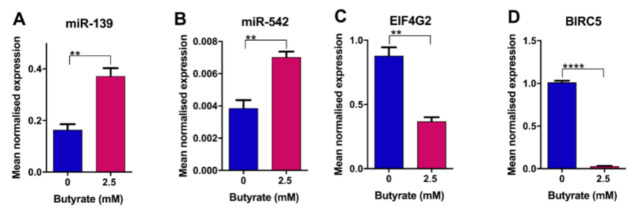
Real-time RT–PCR analysis of networking miRNAs and predicted target gene expression validation in HCT116 cells treated with 2.5 mM butyrate for 24 h. Expression levels of miRNAs and predicted target genes identified by network analysis (**A**) miR-139, (**B**) miR-542, (**C**) *EIF4G2*, (**D**) *BIRC5* in HCT116 cells treated with 0 mM or 2.5 mM butyrate for 24 h. The mean miRNA or mRNA levels ± SEM of (*n* = 3) is represented, and their expression is normalized to *RNU6B* endogenous control (miRNAs only) or the geometric mean of three reference genes, *ACTB*, *B2M* and *GAPDH* (mRNAs only). Significant values are indicated by ** *p* < 0.01, **** *p* < 0.0001. NC = negative control mimic.

Regulation of selected miRNA and target mRNAs by butyrate in CRC cells was examined using Taqman real-time RT-PCR assays. This analysis confirmed that miR-139 (*p* = 0.0053) and miR-542 (*p* = 0.0065) were significantly induced when HCT116 cells were exposed to 2.5 mM butyrate (Figure 6A,B). The validated targets for miR-139 and miR-542 including *EIF4G2* (*p* = 0.0022) and *BIRC5* (*p* < 0.0001), respectively, showed significantly decreased expression after butyrate treatment (Figure 6C,D).

### 2.8. Control of CRC Cell Growth by miRNAs and Butyrate

To determine the effects of miR-139 and miR-542on CRC cell behavior, HCT116 cells were transfected with corresponding miRNA mimics, and changes in the cell cycle, proliferation, and death were assayed. To assess any cooperative or antagonistic effects between the miRNAs and butyrate, the cells were treated 48 h after transfection with or without 2.5 mM butyrate for 24 h. Cell proliferation was measured using the xCELLigence real-time cell imaging system, and cell cycle analysis was performed using flow cytometry.

### 2.9. miR-139 and miR-542 Reduce CRC Cell Proliferation Alone and in Combination with Butyrate

**Figure 7:**
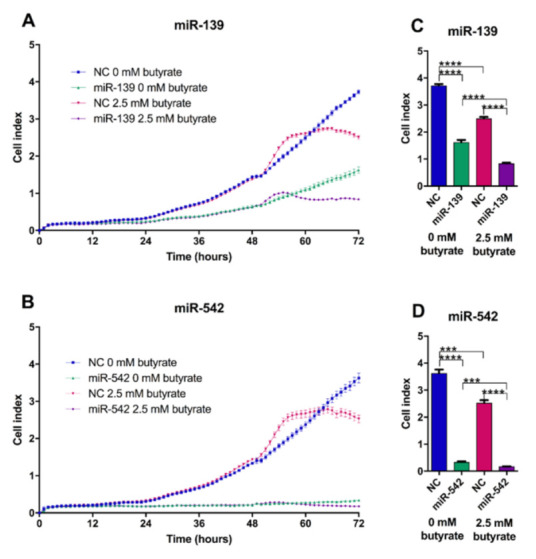
Proliferation of HCT116 cells after transfection with miRNA mimics and butyrate treatment for 24 h. Real-time cell index measurements using the xCELLigence RTCA platform, in HCT116 cells transfected with miRNAs (**A**) miR-139, (**B**) miR-542 for 48 h, followed by 24 h of treatment with 0 mM or 2.5 mM butyrate, over a 72 h post-transfection period. The mean ± SEM (*n* = 4) is shown at 72 h post-transfection (**C**) miR-139, (**D**) miR-542. Significant results are indicated by *** *p* <0.001, **** *p* < 0.0001. NC = negative control mimic.

The proliferation of HCT116 cells was monitored over a 72 h time period post-transfection. Both miR-139 and miR-542 significantly decreased proliferation, both alone and in butyrate-treated conditions (Figure 7). miR-542 showed the most dramatic effects independently of butyrate-producing ~12-fold reduction in cell proliferation (*p* < 0.0001). Butyrate alone significantly decreased proliferation by ~30% in all experiments (comparing NC transfected 0 mM butyrate vs. 2.5 mM butyrate-treated conditions) (*p* < 0.05). The coefficient of drug interaction (CDI) calculations illustrated that both miR-139 and miR-542 act synergistically with butyrate to enhance its antiproliferative properties, with CDIs of 0.77 and 0.74, respectively.

### 2.10. miR-139 and miR-542 Modulate Cell Cycle Alone and in Combination with Butyrate

**Figure 8:**
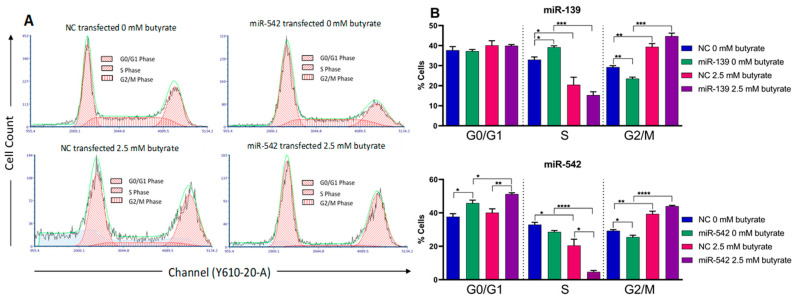
Flow cytometry analysis of the cell cycle in miRNA-transfected HCT116 cells after 24 h of butyrate treatment. (**A**) Examples of flow charts depicting cell cycle analyses of HCT116 cells reverse-transfected with control (NC) or miR-542 mimics for 48 h, followed by 24 h of treatment with 0 mM or 2.5 mM butyrate, over a 72 h post-transfection period. (**B**) Bar charts for cell cycle analysis of HCT116 cells reverse-transfected with miRNA mimics miR-139 or miR-542 for 48 h, followed by 24 h of treatment with 0 mM or 2.5 mM butyrate, over a 72 h post-transfection period. Cells were stained with propidium iodide, and cell percentage measured using the Cytoflex flow cytometer. The mean ± SEM (*n* = 3) is shown. Significant results are indicated by * *p* < 0.05, ** *p* < 0.01, *** *p* < 0.001, **** *p* < 0.0001. NC = negative control mimic.

Cell cycle analysis demonstrated that butyrate alone (e.g., in NC transfected conditions) significantly reduced the percentage of cells in the S phase (*p* = 0.0361) and increased the percentage in the G2/M phase; there was no effect on the G0/G1 phase (Figure 8). Transfection of miR-139 mimics alone slightly but significantly increased the percentage of cells in the S phase and decreased the percentage in the G2/M phase; there was no effect on the G0/G1 phase. When the miR-139 mimic was combined with butyrate, the effect of butyrate on the S phase and G2/M phase predominated and appeared to reverse the effects of the mimics. Transfection of miR-542 mimics alone significantly increased the percentage of cells in the G0/G1 phase and decreased the percentage in the G2/M phase but had no effect on the S phase. When miR-542 was combined with butyrate, again, the effect of butyrate on the S phase and G2/M phase predominated. Interestingly, however, miR-542 and butyrate appeared to synergistically decrease the percentage of cells in the S phase, with the combination giving much greater reduction than either treatment alone (*p* = 0.0152). This result is broadly consistent with the synergistic effect of miR-542 and butyrate on reducing proliferation (Figure 7).

### 2.11. miR-139 and miR-542 Induce CRC Cell Apoptosis Alone and in Combination with Butyrate

To assess the effects of miR-139 and miR-542 on apoptosis, HCT116 cells were transfected with corresponding miRNA mimics and cells treated with butyrate, as previously described. Cells were stained with annexin V and propidium iodide to differentiate between cells in early apoptosis, late apoptosis and necrosis using flow cytometry.

**Figure 9:**
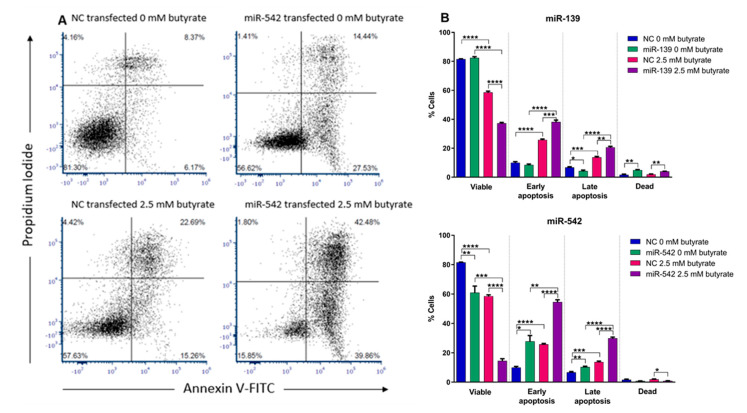
Flow cytometry analysis of apoptosis in miRNA-transfected HCT116 cells after 24 h of butyrate treatment. (**A**) Examples of flow charts depicting the apoptosis analyses of HCT116 cells reverse-transfected with NC or miR-542 mimics for 48 h, followed by 24 h of treatment with 0 mM or 2.5 mM butyrate, over a 72 h post-transfection period. (**B**) Bar charts showing apoptosis analysis of HCT116 cells reverse-transfected with miRNA mimics miR-139 or miR-542 for 48 h, followed by 24 h of treatment with 0 mM or 2.5 mM butyrate, over a 72 h post-transfection period. The mean ± SEM (*n* = 3) is shown. Significant results are indicated by * *p* < 0.05, ** *p* < 0.01, *** *p* < 0.001, **** *p* < 0.0001. NC = negative control mimic.

Butyrate alone significantly reduced the number of viable cells and increased the number of cells in both early and late apoptosis (Figure 9). miR-139 alone had no effect on the total number of viable cells in this assay, although there was a slight but significant reduction in late apoptosis. When combined with butyrate, miR-139 significantly enhanced the capacity of butyrate to reduce the number of viable cells and to induce early (*p* = 0.0008) and late apoptosis (*p* = 0.0015). miR-139 mimics alone produced a slight but significant increase in necrosis (*p* = 0.0047), and this was unaffected by butyrate. The miR-542 mimic alone significantly decreased the percentage of viable cells (*p* = 0.0097) and increased the number of cells in early (*p* = 0.0111) and late apoptosis (*p* = 0.0068) (Figure 9). When combined with butyrate, miR-542 greatly enhanced the capacity of butyrate to reduce the number of viable cells (*p* < 0.0001) and to induce early and late apoptosis (*p* < 0.0001). miR-542 mimics had no clear effect on necrosis. CDI calculations illustrated that both miR-139 and miR-542 showed significantly synergistic behavior in combination with butyrate to further reduce cell viability, with CDIs of 0.63 and 0.33, respectively.

### 2.12. miR-139 and miR-542 Effects are Specific to CRC Cells

CRC is highly heterogeneous, and different CRC cell lines may show varying responses to butyrate. Hence, we sought to confirm the effects of miR-139 and miR-542 on CRC proliferation and survival using a second CRC line, LIM1215, which has a different profile of driver mutations than HCT116. HCT116 cells harbor wild-type (WT) *BRAF* and *TP53* genes and mutant variants of the *KRAS*, *PIK3CA* and *β-catenin* genes. The LIM1215 cell line harbors wild-type *TP53*, *KRAS*, *BRAF* and *PIK3CA* genes and a mutant *β-catenin* gene. We also examined whether these miRNAs could alter the proliferation and survival of noncancerous cells using normal human foreskin fibroblasts (HFF) as a model.

**Figure 10:**
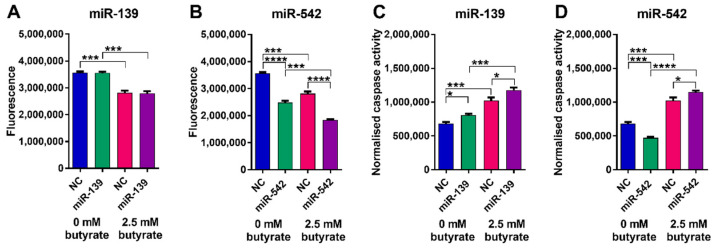
Cell viability and apoptosis in miRNA-transfected LIM1215 cells after 24 h of butyrate treatment. ApoLive-Glo™ multiplex assay: fluorescence and luminescent signals for viability changes (**A**) miR-139, (**B**) miR-542 and normalized caspase activity for apoptosis changes, respectively (**C**) miR-139, (**D**) miR-542 in LIM1215 cells transfected with butyrate-sensitizing miRNAs for 48 h, followed by 24 h of treatment with 0 mM or 2.5 mM butyrate, over a 72 h post-transfection period. Significant results are indicated by * *p* < 0.05, *** *p* < 0.001, **** *p* < 0.0001. NC = negative control mimic.

Butyrate alone significantly reduced the viability of LIM1215 cells and significantly increased apoptosis (*p* < 0.001) (Figure 10). miR-139 did not significantly alter LIM1215 cell viability alone or in combination with butyrate (Figure 10A); however, it did cause a small but significant increase in apoptosis alone and when combined with butyrate (Figure 10B). miR-542 induced a robust and significant decrease in viability alone and when combined with butyrate (*p* < 0.0001) (Figure 10C). Interestingly, miR-542 slightly reduced apoptosis alone; however, it increased apoptosis when combined with butyrate (Figure 10D). Overall, the effects of miR-542 were largely comparable to those observed in HCT116 cells.

**Figure 11:**
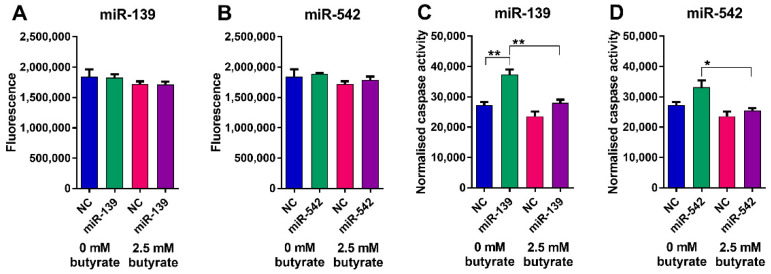
Cell viability and apoptosis in miRNA-transfected HFF cells after 24 h of butyrate treatment. ApoLive-Glo™ multiplex assay: fluorescence and luminescent signals for viability changes (**A**) miR-139, (**B**) miR-542 and normalized caspase activity for apoptosis changes, respectively (**C**) miR-139, (**D**) miR-542 in HFF cells transfected with butyrate-sensitizing miRNAs for 48 h, followed by 24 h of treatment with 0 mM or 2.5 mM butyrate, over a 72 h post-transfection period. Significant results are indicated by * *p* < 0.05, ** *p* < 0.01. NC = negative control mimic.

Butyrate alone had no effect on the number of viable or apoptotic HFF cells (Figure 11). Neither of the miRNA mimics altered the number of viable HFF cells, although miR-139 did increase the number of cells showing caspase activation. Overall, these data suggest that the miRNAs and butyrate have minimal effects on the viability of normal HFF relative to CRC cells.

### 2.13. Validation of miRNA Targets in HCT116 Cells

**Figure 12:**
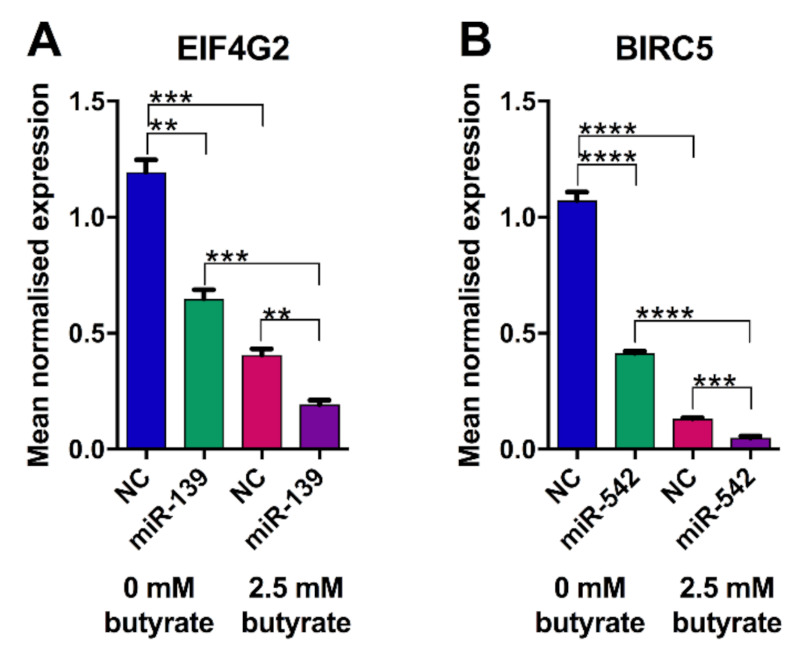
Real-time RT–PCR analysis of miRNA target gene expression in HCT116 cells treated with butyrate for 24 h. mRNA levels of (**A**) *EIF4G2* and miR-542 predicted target gene (**B**) *BIRC5* in HCT116 cells transfected with miRNA or NC mimics for 48 h, followed by 24 h of treatment with 0 mM or 2.5 mM butyrate, over a 72 h post-transfection period. The mean mRNA levels ± SEM (*n* = 3) are represented, and their expression is normalized to the geometric mean of three reference genes, *ACTB*, *B2M* and *GAPDH*. Significant values are indicated by ** *p* < 0.01, *** *p* < 0.001, **** *p* < 0.0001. NC = negative control mimic.

The levels of previously validated miR-139 and miR-542 targets were assessed in HCT116 cells after transfection of miRNA mimics and butyrate treatment. miR-139 alone decreased the expression of its target *EIF4G2* by almost 2-fold (*p* = 0.0011) (Figure 12A). The expression of *EIF4G2* was also reduced ~3-fold by butyrate alone, and the combination of miRNA-139 and butyrate produced ~6-fold repression. miR-542 alone reduced the expression of its target *BIRC5* by ~2.5-fold (*p* < 0.0001). Similar to its effects on *EIF4G2*, butyrate alone repressed expression of *BIRC5* (~8-fold), and the greatest repression was seen with the combination of miR-542 and butyrate (~21-fold). CDI calculations illustrated that both miR-139 and miR-542 act synergistically with butyrate to enhance the reduction in *EIF4G2* and *BIRC5* expression, with CDIs of 0.88 and 0.98, respectively.

To confirm that both miRNA target genes are also expressed in the control HFF cell line (Section 2.12), we compared the abundance of *EIF4G2* and *BIRC5* transcripts in our HCT116 RNA-seq dataset with that in untreated HFF-1 cells, available in GEO accession: GSE93226 [27]. *EIF4G2* showed average abundance, measured in reads per kilobase of the transcript, per million reads mapped (RPKM) of 82.3 in untreated HCT116 cells (*n* = 2), compared with 28.8 in butyrate-treated HCT116 cells (*n* = 2) and 133.2 in untreated HFF cells (*n* = 3). The average *BIRC5* transcript level in untreated HCT116 cells was 38.9 RPKM, which was reduced to 3.15 in butyrate-treated HCT116. HFF-1 cells showed a much lower level of *BIRC5* than untreated HCT116 at 15.8 RPKM, consistent with noncancerous cells expressing this gene at lower levels than cancerous cells. HCT116 RNA-seq data were also consistent with HCT116 RT–PCR data for both target genes.

### 2.14. Silencing of EIF4G2 Alone and in Combination with Butyrate Reduces HCT116 Cell Proliferation

Based on network analysis in Section 2.5, *EIF4G2* had the greatest number of connections with differentially expressed miRNAs, including predicted interactions with miR-146a, miR-146b, miR-3127, and validated interactions with miR-139 and miR-379 (Table 1). Moreover, regulation of *EIF4G2* by miR-139 in CRC cells was confirmed by studies shown in Figure 12. To determine whether *EIF4G2* may be directly involved in CRC proliferation and the response to butyrate, HCT116 cells were transfected with *EIF4G2* or control (NC) siRNA, treated with or without butyrate, and proliferation was measured as previously described.

**Figure 13:**
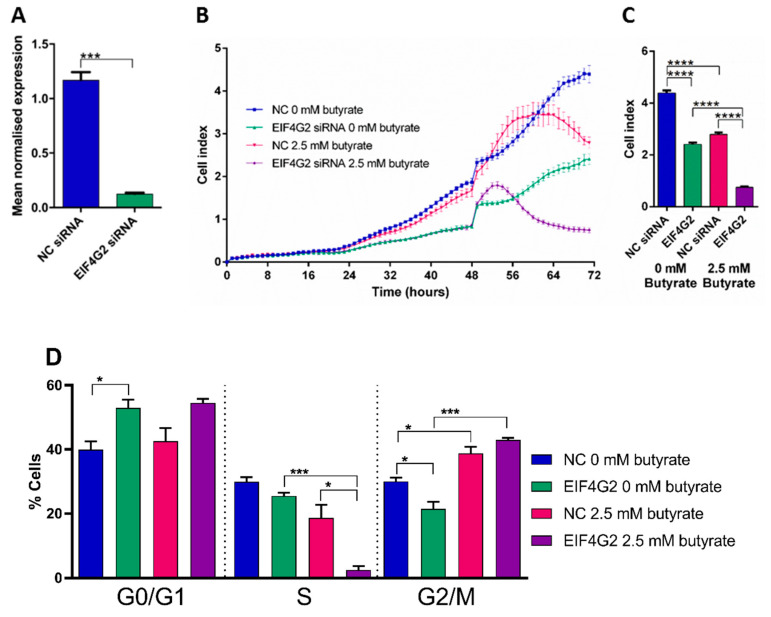
EIF4G2 siRNA knockdown efficiency in HCT116 cells. mRNA levels of EIF4G2 in CRC cells (**A**) HCT116 cells transfected with NC siRNA or EIF4G2 siRNA for 72 h. The mean mRNA levels ± SEM (*n* = 3) are represented, and their expression is normalized to the geometric mean of three reference genes, ACTB, B2M and GAPDH. Real-time cell index measurements using the xCELLigence RTCA platform, in (**B**) HCT116 cells transfected with NC or EIF4G2 siRNA for 48 h, followed by 24 h of treatment with 0 mM or 2.5 mM butyrate, over a 72 h post-transfection period. (**C**) The mean ± SEM (*n* = 4) is shown at 72 h post-transfection with EIF4G2 siRNA. (**D**) Flow cytometry analysis of the cell cycle in siRNA transfected HCT116 cells after 24 h of butyrate treatment. Bar charts showing the cell cycle analysis of HCT116 cells reverse-transfected with EIF4G2 siRNAs for 48 h, followed by 24 h of treatment with 0 mM or 2.5 mM butyrate, over a 72 h post-transfection period. Cells were stained with propidium iodide, and cell percentage measured using the Cytoflex flow cytometer. The mean ± SEM (*n* = 3) is shown. Significant results are indicated by * *p* < 0.05, *** *p* < 0.001, **** *p* < 0.0001. NC = negative control mimic.

The knockdown efficiency of *EIF4G2* siRNA was determined to be ~89% (*p* = 0.0001) (Figure 13A). *EIF4G2* knockdown alone significantly reduced cell proliferation by almost 2-fold (*p* < 0.0001). Butyrate alone reduced proliferation as expected (*p* < 0.0001) (Figure 13B,C). The combination of *EIF4G2* siRNA and butyrate reduced proliferation by ~5-fold, and CDI calculation indicated a significant synergistic effect at 0.49.

Cell cycle analysis showed that *EIF4G2* knockdown alone significantly increased the percentage of cells in G0/G1 phase (*p* = 0.0237) and decreased cells in the G2/M phase (*p* = 0.0251) (Figure 13). Butyrate alone significantly decreased the percentage of cells in the S phase and increased cells in the G2/M phase, as previously shown. The combination of *EIF4G2* knockdown and butyrate synergistically decreased the percentage of cells in the S phase (*p* = 0.0201) and increased cells in the G2/M phase. These data are generally consistent with the effects of *EIF4G2* knockdown and butyrate on cell proliferation.

## 3. Discussion

Butyrate is a well-studied chemo-protective agent with the ability to induce apoptosis, inhibit cell proliferation and regulate the cell cycle in CRC cells through the global regulation of gene expression [10,28]. miRNAs are commonly dysregulated in CRC, and butyrate regulates the expression of key cancer-related miRNAs in CRC, including members of the oncogenic clusters miR-17-92a, miR-106a-363, and miR-106b-25 [12,18,29]. However, the complex miRNA: mRNA interactions and networks that are involved in the butyrate response of CRC cells remain to be fully elucidated. Here we identified miRNAs and mRNAs that are regulated by butyrate in CRC cells and applied a systems biology approach to identify miRNA-target interactions that may be involved in butyrate’s anticancer effects. This analysis identified several butyrate-sensitive miRNAs: mRNA pairings that are predicted to be important in cell cycle regulation and apoptosis in CRC cells. Moreover, we functionally characterized two miRNAs, miR-139 and miR-542, that are regulated by butyrate and can also synergize with butyrate to enhance its anticancer properties.

Butyrate was found to alter the expression of thousands of protein-coding and miRNA genes in CRC cells. Butyrate is a HDACi known to alter gene expression through H3 and H4 hyperacetylation [28], leading to a more open chromatin configuration and facilitating transcription [30]. The finding that similar numbers of genes were upregulated and downregulated by butyrate confirms previous findings in CRC cells [10]. Similarly, miRNA profiling data obtained by massively parallel sequencing are consistent with earlier miRNA microarray profiling studies examining CRC butyrate responses, which previously highlighted changes in the expression of the paralogous miR-17~92 and miR-106a~363 clusters [12,18,27]. The mechanisms underlying downregulation have been studied in CRC, and hepatocellular carcinoma cells, whereby histone acetylation around promoter regions, and specifically transcription start sites of particular genes, can decrease, resulting in reduced gene expression [31]. GO analysis of these butyrate-downregulated genes revealed enrichment of genes involved in cell proliferation [31]. Butyrate can also regulate non-coding RNAs, transcription factors and heterochromatin factors, which could indirectly mediate repression of gene expression [11,32].

Alternative splicing analyses revealed that butyrate treatment predominantly promotes exon skipping in CRC cells. Exon array analyses in Hela cells previously demonstrated that butyrate could modulate alternative splicing of ~700 genes primarily involved in the cell cycle, apoptosis and transcription regulation [33]. Notably, exon skipping was found to increase after butyrate treatment due to increased H4 acetylation and accelerated RNA polymerase II elongation rate within alternatively spliced regions [33,34]. Our work provides more evidence for the role of butyrate in RNA processing and has revealed an even greater number of alternatively spliced genes suggesting that butyrate controls cell behavior by diversifying the proteome.

In the present study, GO analysis of butyrate-regulated DEGs in CRC cells showed enrichment for genes involved in the cell cycle, as well as DNA related functions. This was consistent with previous studies of butyrate-regulated DEGs that showed functional enrichment of genes in pathways such as regulation of mismatch repair, cell cycle, and DNA replication [35]. Further pathway enrichment analysis revealed that the DEGs identified were enriched in the cell cycle, DNA replication and apoptosis pathways. Again, this is consistent with previous analyses of the butyrate response using microarray or small-scale profiling methods [10,36].

We used integrative network construction to identify 24 negatively correlating miRNA: mRNA interactions potentially relevant in the butyrate response in CRC cells. In this network, EIF4G2 was a key hub with the greatest number of connections, including five interactions with miRNAs: hsa-miR-139-5p, hsa-miR-146a-5p, hsa-miR-146b-5p, hsa-miR-3127-5p and hsa-miR-379-5p. We selected miR-139 and miR-542 for further study because they had been previously defined as tumor suppressor miRNAs with possible oncogenic target genes in CRC and other cancers. However, other miRNAs with two or more target gene interactions would also be valuable to study in future work.

miR-542 is a tumor suppressor reported to impact the development and progression of cancers such as CRC, hepatocellular carcinoma and breast cancer, where it is typically downregulated [37,38,39,40]. The miR-542-target gene *BIRC5* (survivin) is a well-studied member of the inhibitor of apoptosis (IAP) family that is highly expressed in several cancer types and is involved in the regulation of numerous cellular pathways such as apoptosis, cell cycle, proliferation, metastasis and invasion [41,42]. *BIRC5* was previously shown to be regulated by miR-542 in CRC [38,43]; moreover, miR-542 was found to decrease proliferation and induce apoptosis in CRC cell lines [37,39]. A direct interaction between miR-542 and the *BIRC5* 3′UTR in CRC cells has already been validated in vitro [36]. Our study has extended on this prior knowledge, revealing a new role for miR-542 in the butyrate response. We found that miR-542 is induced by butyrate and that it synergistically enhances the ability of butyrate to reduce proliferation and induce apoptosis. Moreover, we found that miR-542 and butyrate have synergistic effects on the cell cycle, increasing cells in G0/G1 and reducing cells in the S phase. Consistent with this finding, miR-542 was previously reported to inhibit hepatocellular carcinoma and lung cancer cell proliferation by inducing G1 phase arrest [44,45].

miR-139 is commonly downregulated in many cancer types such as CRC, esophageal cancer, lung cancer and acute myeloid leukemia (AML) and is considered to be a tumor suppressor in these contexts [46,47,48,49,50]. In the present study, we showed for the first time that miR-139 is a butyrate-regulated miRNA that also acts synergistically with butyrate to reduce proliferation and enhance apoptosis of CRC cells. Our results are also consistent with a previous report that miR-139 alone can decrease the growth of HCT116 cells [51], although this response can be variable [50,52] and is possibly also reflected in the differential response seen in LIM1215 cells. Our HCT116 cell cycle analysis demonstrated that the combination of miR-139 and butyrate induces cells to arrest in the G2/M phase, which may contribute to reduced proliferation. This is in contrast to other studies indicating that miR-139 can promote G0/G1 phase arrest of HCT116 cells and prostate cancer cells by induction of G1 phase inhibitors [51,53]. These differences may be methodological or relate to subtle differences in cell lines.

*EIF4G2* was previously identified as a direct target of miR-139 in AML, with luciferase assays identifying binding sites in both the 3′UTR and 5′UTR, while in glioblastoma, only the 3′UTR binding site was validated with reporter assays and also anti-Ago2-mediated RNA-immunoprecipitation assays [46,54]. The role of EIF4G2 in CRC is not well studied, although it is reported to control the G1 phase inhibitor p27^Kip1^ to alter proliferation in glioblastoma, human embryonic kidney and AML cells [46,55]. EIF4G2 showed the greatest number of connections with butyrate-regulated, differentially expressed miRNAs in our network analysis, suggesting that it may have an important role in the butyrate response of CRC cells. *EIF4G2* inhibition alone significantly reduced CRC cell proliferation, and this was synergistically enhanced in the presence of butyrate. This phenocopied the effects of transfecting miR-139 mimics with and without butyrate treatment in CRC cells. In contrast, the effects of *EIF4G2* siRNAs on the cell cycle did not completely phenocopy the effects of miR-139. *EIF4G2* siRNAs alone increased the percentage of cells in the G0/G1 phase, whereas miR-139 alone had no effect. The combination of *EIF4G2* siRNA and butyrate-reduced cells in the S phase; however, this effect was not seen with the combination of butyrate and miR-139 mimics. The differences observed between the siRNA and miRNA mimic-induced responses may be because the miR-139 mimic only induced a ~50% knockdown of *EIF4G2* compared to nearly 90% for *EIF4G2* siRNA. The differences may also relate to the ability of miR-139 to modulate targets in addition to *EIF4G2* in CRC cells; such target genes could be investigated in future studies. The mechanism(s) underlying the synergistic response between miRNA mimics and butyrate remains to be determined, but it likely involves cooperation between the miRNAs and other butyrate-regulated effectors, the latter potentially including other miRNAs or transcription factors.

Overall, this study provides greater insight into the network of miRNA: mRNA interactions that are involved in the butyrate response in CRC cells. It further identified specific tumor suppressor miRNAs that are induced by butyrate in CRC cells and function synergistically with butyrate to control cell growth and survival. The potential for these miRNAs to have therapeutic anticancer applications, in combination with HDAC inhibitors, should be further tested in preclinical models.

## 4. Materials and Methods

### 4.1. Cell Culture

Human HCT116 colorectal carcinoma cells and HFF foreskin fibroblasts were acquired from ATCC (Manassas, VA, USA). Human LIM1215 colorectal carcinoma cells were acquired from Sigma-Aldrich (St. Louis, MO, USA). Cells were maintained at 37 °C and 5% CO_2_ in McCoy’s 5A (Modified) medium (HCT116) (Invitrogen, Thermo Fisher Scientific, Carlsbad, CA, USA) or Dulbecco’s modified Eagle’s medium 1X (HFF and LIM1215) (Invitrogen) containing 10% fetal bovine serum (Bovogen Biologicals, Victoria, Australia). Cells were grown to <85% confluence and mycoplasma-free.

### 4.2. Total and Small RNA-Seq

Total RNA expression was determined using RNA from HCT116 cells (*n* = 2), and small RNA-seq was determined using RNA from HCT116 cells (*n* = 3) untreated (0 mM) and butyrate-treated (2.5 mM) cells over 48 h. RNA was extracted using TRIzol^®^ Reagent (Invitrogen) as per the manufacturer’s instruction. Prior to sequencing preparation, samples were analyzed for quality using the Agilent 2100 Bioanalyzer system and Agilent RNA 6000 Nanochip (Agilent Technologies, Santa Clara, CA, USA). The RNA-Seq libraries were prepared and sequenced at the Flinders Genomics Facility (Flinders University, Adelaide, Australia). Samples underwent rRNA depletion (only total RNA-seq), adapter ligation and PCR amplification using the TruSeq Stranded Total RNA sample preparation kit (Illumina Inc., San Diego, CA, USA) for total RNA-seq and TruSeq small RNA library preparation (Illumina Inc.) for small RNA-seq, as per manufacturer’s instructions. For total RNA-seq, aliquots (1 μg) of the 4 RNA samples (duplicate samples for the control and untreated experimental groups) were subjected to paired-end 100 bp sequencing using the Illumina NextSeq sequencing platform (Illumina Inc.), and approximately 30 million reads were generated per sample. While 6 RNA samples (triplicate samples for the control and untreated experimental groups) for small RNA-seq generated approximately 10 million reads each.

The QIAseq^TM^ Targeted RNA Panel (human apoptosis and cell death pathway finder RHS-002Z) (Qiagen, Hilden, Germany) was used as a complementary method to determine gene expression. RNA was collected from HCT116 cells (*n* = 2) untreated (0 mM) and butyrate-treated (2.5 mM) cells over 48 h, as mentioned previously. Samples were processed as per the manufacturer’s protocol (Qiagen) and sequenced using the Illumina MiSeq sequencing platform. Approximately 19.5 million reads were generated.

**Figure 14:**
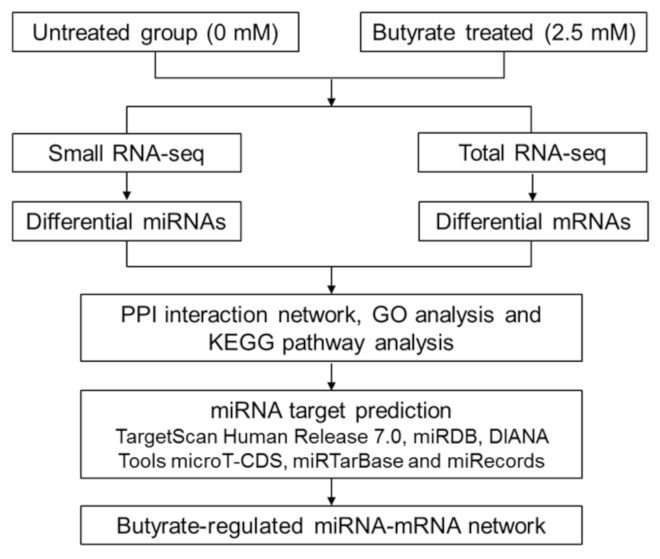
Flow chart of miRNA-mRNA network construction associated with the butyrate response in CRC cells. PPI network, protein–protein interaction network; GO analysis, gene ontology analysis; KEGG pathway analysis, Kyoto Encyclopedia of Genes and Genomes.

RNA-seq data were analyzed by the Flinders Genomics Facility (Flinders University, Adelaide, Australia). Data were trimmed for adaptors using the Trimmomatic [56] program, followed by a quality analysis of reads using FastQC and assembly, mapping and alignment of reads to Ensembl human genome (Grch38.p5_v24) using STAR [57]. Aligned reads were then converted to raw counts using HTSeq [58], and differential expression analysis was performed using DESeq2 [59]. QIAseq data were analyzed using the GeneGlobe Data Analysis Center (Qiagen) online tool to perform quality analysis of reads, normalization and molecular tag count. The work-flow for RNA sequencing and network construction is presented in Figure 14.

### 4.3. Alternative Splicing Analysis

Replicate multivariate analysis of transcript splicing (rMATS) data analyses were performed. Criteria used to select significant changes in splicing events included False Discovery Rate (FDR) <0.05 and Percent Spliced-in Index (PSI) ≥0.1. rMATS version 4.1.0 [60] was used in this study.

### 4.4. Network Construction and Pathway Analysis

#### 4.4.1. Protein–Protein Interaction (PPI) Network Construction

miRNA-mRNA networks were produced from the top differentially expressed miRNAs and mRNAs selected from small RNA-seq and total RNA-seq data, respectively. Criteria used to select differentially expressed miRNAs included log2FC < −1 or log2FC > 1, adjusted *p* < 0.05 and average raw counts for selected miRNAs >4 reads per million. Criteria used to select differentially expressed mRNAs included log2FC < −1.5 or log2FC > 1.5, adjusted *p* < 0.01 and average raw counts for selected mRNAs >0.7 reads per million. The differing fold-change criteria between miRNAs and mRNAs reflect the different biological roles of the two RNA types, with miRNAs often showing significant downstream effects following small changes while also limiting the number of molecules to generate tractable networks. The final protein-coding gene list was refined using the biological network analysis and visualization tool, NetworkAnalyst (http://www.networkanalyst.ca; accessed on 19 January 2018) [61,62], to define interactors with degree interactions (node connections) >1. The PPI database, IMEx Interactome, which is a comprehensive literature-curated database from InnateDB, was used to define the network. Only zero-order networks were investigated as these show direct interactions with the input list (seed proteins). The protein–protein interaction network was visualized using the open-source network construction software, Cytoscape (Version 3.4.0) [63] and then analyzed (undirected network) using the in-built Network Analyzer tool to determine the network properties.

#### 4.4.2. Gene Ontology (GO) Analysis

Gene ontology (GO) enrichment analysis was used to examine dysregulated genes identified in the PPI network. ClueGO [64] is a Cytoscape plug-in that can be used for GO enrichment analysis of gene lists. ClueGO was used to identify the enriched GO terms for dysregulated mRNA genes for the classification: Biological Process, Cellular Compartment and Molecular Function.

#### 4.4.3. Pathway Enrichment Analysis

ClueGO pathway enrichment analysis was used to further investigate the role of the differentially expressed protein-coding genes in the butyrate response of CRC cells. KEGG pathway analyses were utilized to identify key canonical signaling pathways and associated genes.

#### 4.4.4. miRNA-mRNA Network Construction

miRNA-mRNA networks were then constructed based on miRNA target predictions. miRNA target predictions were collated from multiple target prediction programs including TargetScan Human Release 7.0 (total context++ score ≤−0.3) [21], miRDB (prediction score ≥85) [22], DIANA Tools microT-CDS (miTG score ≥0.95) [23], miRTarBase (strong evidence) [24,25] and miRecords (strong evidence) [25]. The Cytoscape application, CluePedia [64], was used to perform network construction for differentially expressed mRNAs and miRNAs, including those that were predicted or validated by two or more programs or databases, respectively and only those with predicted interactions and with anti-correlating expression values. The rationale behind this is that if a miRNA targets an mRNA, its levels are very likely to be reduced due to target degradation. If the miRNA expression is reduced, then the mRNA target expression is expected to increase and vice versa. Gene ontology analysis and literature review was then used to further refine the list and identify key relevant interactions in colorectal cancer for further investigation.

#### 4.4.5. Transcription Factor Enrichment Analyses

Transcription factor enrichment analysis of RNA-seq data, using ChEA3 [65], was employed to predict associations between altered TF expression and differential expression of protein-coding genes. The TransmiR v2.0 database [66] that predicts miRNA targets of TFs was interrogated to further investigate the possible influence of ChEA3-identified transcription factors on miRNA gene transcription.

### 4.5. Reverse Transfections and Treatments with miRNA Mimics and Butyrate

Proliferation experiments were performed using 16 well E plates (ACEA Biosciences, San Diego, CA, USA) and the xCELLigence Real-Time Cell Analysis (RTCA) Dual Purpose (DP) platform (ACEA Biosciences). Reverse-transfections were performed using Llipofectamine 2000 (Invitrogen) to deliver miRNA mimics, or negative control (NC) mimics (a scrambled sequence with no reported biological significance) (GenePharma, Shanghai, China) and target gene siRNAs or NC siRNAs (Qiagen) to CRC cells. All transfections were performed in triplicate or quadruplicate per miRNA mimic per treatment group. Prior to transfection, xCELLigence E plates were blanked using 50 μL of the growth medium, contributing to part of the total well volume of 150 μL; 50 μL of growth medium was also added to 96-well plates prior to transfections to maintain consistency in Incucyte studies, although not required for blanking. Transfections were performed using 0.25 μL of lipofectamine 2000 in 25 μL of Opti-MEM per well which was combined with 0.15 μL of 20 µM mimic in 25 μL of Opti-MEM per well. The final concentration of miRNA and NC mimics in each well was 20 nM each. Cells were diluted and seeded at 7500 cells in 50 μL of growth media per well in 16 well E plates or 96-well plates. The total well volume was 150 μL. Plates were incubated at RT for 20 minutes before being placed in the xCELLigence instrument (cell proliferation studies) or Incucyte (apoptosis studies) for a 48 h post-transfection period at 37 °C and 5% CO_2_.

Reverse-transfections were also performed using Greiner 24 well plates (Greiner Bio-One) to generate enough cells for RNA or protein extraction. These transfections involved using 1 μL of lipofectamine 2000 in 50 μL of Opti-MEM per well which was combined with 0.6 μL of 20 µM mimic (stock) in 50 μL of Opti-MEM per well. Cells were seeded at 75,000 per well in 500 μL of the growth medium, to make a total well volume of 600 μL. Cells for the RNA-seq and flow cytometry (cell cycle and apoptosis) were collected from 6 well plates (Greiner Bio-One). These transfections involved using 3 μL lipofectamine 2000 in 250 μL of Opti-MEM per well, which was combined with 2 μL of mimic in 250 μL of Opti-MEM per well. Cells were seeded at 200,000 per well in 1.5 mL of the growth medium to make a total well volume of 2 mL.

CRC cells were exposed to 0 mM or 2.5 mM sodium butyrate (Sigma-Aldrich) treatment after the 48 h transfection period. Sodium butyrate powder was dissolved in the appropriate volume of McCoy’s 5A (modified) medium to make a 1 M solution of butyrate. The solution was filtered using a 0.2 µM filter and a 2 mL syringe. To avoid disturbing cells, the butyrate solution was diluted to 5 mM in growth medium and used to replace half of the total volume in each well to reach a final concentration of 2.5 mM.

### 4.6. RNA Extraction and Real-Time RT–PCR

#### 4.6.1. microRNA Real-Time RT–PCR

Total RNA was extracted using TRIzol^®^ Reagent (Invitrogen) as per the manufacturer’s instruction. RNA was quantified using the Nanodrop8000 spectrophotometer (Thermo Fisher Scientific). miRNA expression was determined using RNA from control and butyrate-treated (2.5 mM) CRC cells. cDNA was synthesized from 20–100 ng total RNA using microRNA- or RNU6B (endogenous control)-specific primers as specified by the TaqMan MicroRNA assay protocol (Thermo Fisher Scientific). Real-time RT–PCR was performed as specified by the TaqMan MicroRNA assay protocol in triplicate (Thermo Fisher Scientific), using miRNA-specific primers (assay IDs: miR-139-5p: 005364_mat and miR-542-3p: 001284). Thermocycling was performed using a Qiagen Rotorgene Q (Qiagen). Expression levels were calculated from Ct values using Qgene [67] and normalized against the endogenous small nuclear RNA gene, RNU6B (assay ID: 001093).

#### 4.6.2. mRNA Real-Time RT–PCR

For mRNA expression analysis, total RNA was DNase I -treated (DNA-free; Promega, Madison, Wisconsin, USA). cDNA was created from 1 µg of total RNA using Random Primer 6 (New England Biolabs, Ipswich, MA, USA), M-MLV reverse-transcriptase enzyme (Promega) and RNase H minus (Promega). Real-time RT–PCR was performed according to Power SYBR green protocol (Thermo Fisher Scientific) using the following primers: BIRC5 (forward): 5′ACTGAGAACGAGCCAGACTTG3′ and BIRC5 (reverse): 5′TGTTCCTCTATGGGGTCGTCA3′; EIF4G2 (forward): 5′TGTTCCAGGTGAATCAGTGGC3′ and EIF4G2 (reverse): 5′GCAGTGGTTAGGTCAAATGCAG3′ (Integrated DNA Technologies, Singapore), using triplicate reactions. Thermocycling was performed using a Qiagen Rotorgene Q (Qiagen). Results were normalized relative to the geometric mean of 3 endogenous controls including GAPDH (forward): 5′TGCACCACCAACTGCTTAGC3′ and GAPDH (reverse): 5′GGCATGGACTGTGGTCATGAG3′, B2M (forward): 5′GCCGTGTGAACCATGTGACTTT3′ and B2M (reverse): 5′CCAAATGCGGCATCTTCAAA3′ and ACTB (forward): 5′TTGCCGACAGGATGCAGAAG3′ and ACTB (reverse): 5′GCCGATCCACACGGAGTACT3′ (Sigma-Aldrich).

### 4.7. Flow Cytometry

#### 4.7.1. Cell Cycle Analysis

For cell-cycle analysis, cells were spun at 300 g for 5 minutes and resuspended in 1 mL of 1× PBS in a 1.5 mL microcentrifuge tube. The pellet was resuspended in 200 μL of 1× PBS by vigorously pipetting several times to create a single-cell suspension. Cells were vortexed, and 800 μL of 100% cold ethanol added dropwise to get a final concentration of 80% ethanol. Samples were transferred to −20 °C for 2 h and then immediately centrifuged at 300× g for 10 minutes. Cells were washed with 1 mL of cold 1× PBS and spun at 600 g for 10 minutes at 4 °C. Cells were resuspended in 400 μL staining solution (PI/RNAse/Triton-x) and passed through cell strainer FACS tube tops (Thermo Fisher Scientific). Cells were incubated for 30 minutes in the dark at RT and analyzed or stored at 4 °C and read within 48 h.

#### 4.7.2. Cell Death Analysis

For cell death analysis, cells were centrifuged at 10,000 rpm for 5 min at 4 °C. The media was removed, and the pellet resuspended in 1 mL 1× PBS. Centrifugation and resuspension steps were repeated once. Pellets were resuspended in 100 μL 1× annexin V-binding buffer (BD Biosciences, New South Wales, Australia). To each tube, 2 μL of annexin V (BD Biosciences) and 5 µL of propidium iodide (PI) (BD Biosciences) were added. Tubes were gently vortexed and incubated for 15 minutes at RT in the dark. After incubation, 100 μL of 1× binding buffer was added to each tube. Tubes were stored on ice and analyzed within 1 h.

### 4.8. Data Analysis

Data were graphically presented using GraphPad Prism (GraphPad Software Inc., San Diego, CA, USA) and statistically analyzed using unpaired *t*-tests, with a *p* value < 0.05 indicating statistical significance. Graphs display the mean of at least *n* = 3 ± standard error of the mean (SEM).

The coefficient of drug interaction was calculated for xCELLigence proliferation data using the calculation CDI = AB/(A × B), whereby A = miRNA mimic/inhibitor/siRNA to negative control ratio, B = 2.5 mM butyrate treatment to negative control, AB = combination of miRNA mimic/inhibitor/siRNA and 2.5 mM butyrate treatment to negative control ratio [68]. CDI < 1, = 1 or > 1 indicates that the drugs are synergistic, additive or antagonistic, respectively and a CDI < 0.7 indicates that the drug is significantly synergistic [68].

## 5. Conclusions

In summary, this study has revealed a novel butyrate-regulated RNA-interaction network in CRC cells and the ability of miRNAs to enhance the anticancer properties of butyrate. Butyrate-sensitizing miRNAs were found to further reduce CRC cell proliferation, induce apoptosis and regulate the cell cycle. Silencing of a hub target gene, *EIF4G2*, was revealed to significantly enhance the anticancer effects of butyrate on CRC cells. There are several future directions for this work, including further investigating other key interactions that were identified during integrative network analysis, such as members of the oncogenic miR-17-92 cluster, miR-18a and miR-19a, which are known to have key roles in CRC development and progression [69,70,71]. While *EIF4G2* seems to be a promising target, its effects on CRC cell growth and death will need to be further elucidated in cell studies and more physiologically relevant 3D models such as organoids before proceeding to animal studies [72]. While further investigation is necessary, this study may provide the basis to develop these miRNAs and this gene as potential therapeutic targets.

## Figures and Tables

**Table 1:** Cell-cycle-related miRNA-mRNA interactions were identified by interactive network analysis. miRNA-mRNA predicted and validated interactions collated from cell cycle network analysis, which were identified in two or more programs or databases. V = validated targets from miRTarBase or miRecords, V (literature) = validated targets that did not appear in miRTarBase or miRecords but were found to be validated in the literature or (-) representing unvalidated targets.

miRNAs	Expression	Genes	Expression	Program/Database	Validated
hsa-miR-542-3p	Up	BIRC5	Down	TargetScan, miRTarBase	V
hsa-miR-532-3p	Up	BORA	Down	microT-CDS, TargetScan	-
hsa-miR-503-5p	Up	CDC25A	Down	miRTarBase, miRecords	V
hsa-miR-424-5p	Up	CHEK1	Down	miRDB, miRTarBase	V
hsa-miR-18a-5p	Down	CTGF	Up	miRDB, miRTarBase	V
hsa-miR-200b-3p	Down	DUSP1	Up	microT-CDS, miRDB	-
hsa-miR-200c-3p	Down	DUSP1	Up	microT-CDS, miRDB	V (literature)
hsa-miR-139-5p	Up	EIF4G2	Down	microT-CDS, miRDB	V (literature)
hsa-miR-146a-5p	Up	EIF4G2	Down	microT-CDS, miRDB	-
hsa-miR-146b-5p	Up	EIF4G2	Down	microT-CDS, miRDB	-
hsa-miR-3127-5p	Up	EIF4G2	Down	microT-CDS, miRDB	-
hsa-miR-379-5p	Up	EIF4G2	Down	microT-CDS, miRDB	V (literature)
hsa-miR-222-3p	Down	ETS1	Up	microT-CDS, miRTarBase	V
hsa-miR-532-3p	Up	HMGA2	Down	miRDB, TargetScan	-
hsa-miR-200b-3p	Down	JUN	Up	microT-CDS, miRDB	V (literature)
hsa-miR-200c-3p	Down	JUN	Up	microT-CDS, miRDB	V (literature)
hsa-miR-381-3p	Up	KIF11	Down	microT-CDS, miRDB	-
hsa-miR-424-5p	Up	KIF23	Down	miRDB, TargetScan, miRTarBase	V
hsa-miR-135b-5p	Down	LZTS1	Up	microT-CDS, miRDB, miRTarBase	V (literature)
hsa-miR-335-3p	Down	PRKAA2	Up	microT-CDS, miRDB	-
hsa-miR-19a-3p	Down	RHOB	Up	microT-CDS, miRDB, TargetScan	V (literature)
hsa-miR-542-3p	Up	UBE2E1	Down	microT-CDS, miRDB	-
hsa-miR-381-3p	Up	WEE1	Down	microT-CDS, miRTarBase	V
hsa-miR-424-5p	Up	WEE1	Down	microT-CDS, miRDB, TargetScan, miRTarBase	V

## Data Availability

Datasets will be made available in a publicly accessible repository. Publicly available datasets were analyzed in this study. The data can be found at https://www.ncbi.nlm.nih.gov/geo/query/acc.cgi?acc=GSE93226.

## Supplementary Materials

The following are available online at https://www.mdpi.com/2072-6694/13/4/636/s1, Figure S1: Correlation analysis between Illumina Total RNA-seq and QIAseq Targeted RNA panel., Table S1: Differentially expressed miRNAs, Table S2: Differentially expressed mRNAs, Table S3: Transcription factor analyses, Table S4: PPI_network_interactors. Table S5: Integrative network interactions.
