# Supplementary material for: Integrative Transcriptomic Network Analysis of Butyrate Treated Colorectal Cancer Cells
Source: Cancers (Basel). 2021 Feb 5;13(4):636. doi: 10.3390/cancers13040636 (PMC7914650; doi:10.3390/cancers13040636)
Supplement: Supplementary file 1 [file cancers-13-00636-s001.zip › cancers-1066397-supplementary-update/Figure S1. Correlation analysis between Illumina Total RNA-seq and QIAseq Targeted RNA panel..docx]

Integrative transcriptomic network analysis of butyrate treated colorectal cancer cells

**Saira R. Ali, Ayla V. Orang, Shashikanth Marri, Ross A. McKinnon, Robyn Meech and Michael Z. Michael**


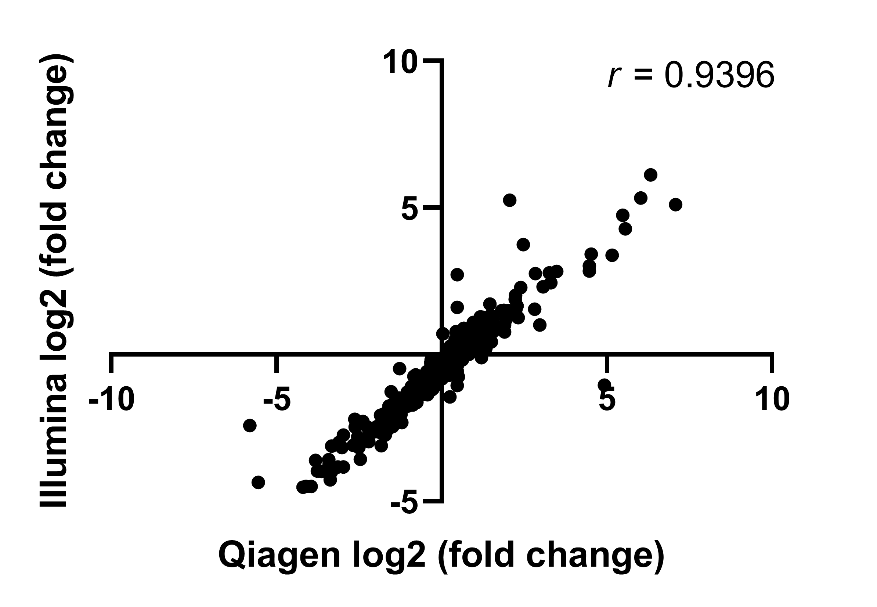


**Figure S1.** Correlation analysis between Illumina Total RNA-seq and QIAseq Targeted RNA panel.
